# Lung "holes" after cryobiopsy: a case report

**DOI:** 10.1186/s12890-021-01640-1

**Published:** 2021-08-23

**Authors:** Roberto Piro, Sofia Taddei, Matteo Fontana, Chiara Scelfo, Eleonora Casalini, Nicola Facciolongo

**Affiliations:** Pulmonology Unit, Azienda Unità Sanitaria Locale - IRCCS di Reggio Emilia, Via Amendola 2, 42123 Reggio Emilia, Italy

## Abstract

**Background:**

Transbronchial lung cryobiopsy is a safe technique increasingly used in the study of lung diseases. Until now, only a case of pneumatocele was described but this interesting condition is probably underestimated because CT scan is routinely not performed after transbronchial lung cryobiopsies.

**Case presentation:**

We report a case of a woman presenting two pneumatoceles after lung cryobiopsies performed for the study of an interstitial lung disease. The finding was obtained with a CT scan performed because of the appearance of hemoptysis, four days after the biopsies.

**Conclusions:**

Small cavitations could develop after cryobiopsies in the absence of an active infection. Studies that prospectively perform CT scan of the chest in patients who have undergone these samplings could be useful to know the incidence of iatrogenic lesions.

## Background

In the last years, transbronchial lung cryobiopsies (TBLC) has emerged as a useful technique for the diagnosis of interstitial and other lung diseases [1]. Indeed, if properly performed, cryobiopsy is generally safer than surgical lung biopsy with lower contraindications, side effects, costs and hospitalization time [2, 3]. Recently, a case of a 69-year-old man subjected to TBLC that presented transient pulmonary cavitations with surrounding rims and fading ground glass infiltrates was reported [4]. CT scan was performed the day after the bronchoscopy because of chest pain. The histopathological exam of the biopsies showed nonspecific interstitial pneumonia (NSIP) with mixed dust nodules. This paper was the first reporting cavitated lesions after TBLC in non-transplanted patients. To date, the only similar cases concern lung abscesses [5] or large excavated lesions [6, 7]. Thus far, uniquely a lung transplanted population was studied in terms of incidence, evolution and characteristics of CT lesions following TBLC [8]. The general frequency of cavitated lesions after TBLC is unknown because CT scan is routinely not performed in the days after TBLC, so it is possible that changes occurring in the lung parenchyma after these biopsies may be unnoticed.

## Case presentation

**Fig. 1:**
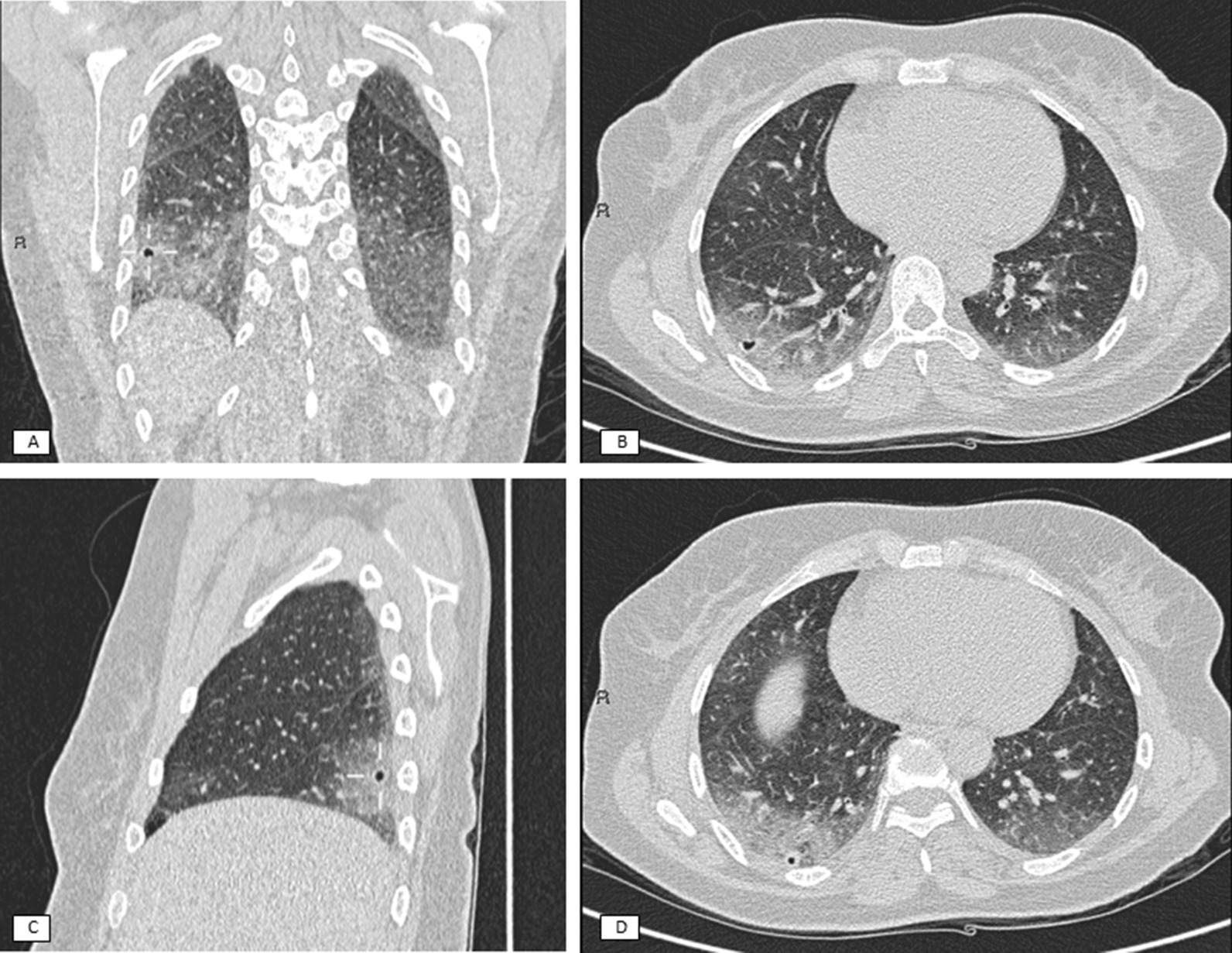
CT scan five days after cryobiopsies. **a** Lateral pneumatocele (arrows) in coronal plane. **b** Lateral pneumatocele in sagittal plane. **c** Lateral pneumatocele (arrows) in axial plane. **d** Medial pneumatocele in axial plane

A 52-year-old female non-smoker patient was referred to our department because of cough and progressive dyspnea for over six months. She had a medical clinical history of psoriasis and primary biliary cholangitis. Physical examination revealed fine crackles bilaterally and was otherwise unremarkable. Lung function tests showed reduced diffusion capacity (52% of predicted value) while the other parameters resulted normal. Chest CT scan showed bilateral ground glass opacities in both lungs, especially in the lower lobes. On the recommendation of the multidisciplinary ILD board, the patient underwent TBLC, using a flexible bronchoscope inside a rigid tracheoscope. Fluoroscopy was used to guide the sampling: using a 1.9 mm cryobiopsy probe and freezing it for 5 s, three biopsies were obtained in the lateral segment and one in the posterior segment of the right lower lobe. There was no immediate complication and, in particular, no bleeding occurred. The histological exam of the TBLC showed large fragments of lung parenchyma, diffusely involved by a mixed (fibrosing and cellular) NSIP pattern. Four days after the sampling, the patient presented with hemoptysis and she underwent the chest CT scan, revealing two cavitated lesions in the sampled sites (Fig. 1); they were absent in the previous scan. The lesions had a thin and regular wall, consisting with pneumatoceles [9]. The greater of them was 6 × 4 mm and the smaller was 3 × 4 mm. These lesions were surrounded by ground glass areas, to a greater extent than was previously present; this was probably due to the addition of bleeding to the pre-existing manifestation of the underlying disease. The patient did not show signs of infection and she no longer presented hemoptysis so she was discharged without antibiotic therapy or prescription for further CT scan for this reason.

## Discussion and conclusions

Generally, lung cavitation of acute/subacute onset are considered to be caused by infectious agents [10]. The unique published study evaluating the incidence, evolution and characteristics of CT lesions following TBLC was conducted in lung transplantated patients [8]. After obtaining 112 cryobiopsy samples, the authors observed 46 opacities greater than 10 mm, including ground-glass, solid, cavitated or a combination of these lesions. Being a transplanted population, the findings of this study may not be traceable to other patients because of specific factors that could be involved, such as toxicity to immunosuppression or post-transplantation alteration of the lymphatic drainage. In the prospective follow up, most cavitated opacities resolved or evolved to solid residual opacities; only two cavitations remained unchanged after 3 and 6 months.

Our report reveals that little cavitations could develop after transbronchial lung cryobiopsy in the absence of an active infection, likely as a transient consequence of the trauma caused by the sampling, even if the pathogenic mechanism has not been investigated. The frequency of these conditions is probably underestimated. With the increasing use of this sampling technique, the reporting of findings like these becomes even more necessary. Even if in clinical practice CT scan after the biopsies should be performed only in case of new symptom onset, studies that prospectively perform CT scan of the chest in patients who have undergone cryobiopsies could be useful to understand their incidence.

## Data Availability

The datasets used and analyzed during the current study are available from the corresponding author on reasonable request.

## Abbreviations

CT: Computed tomography
ILD: Interstitial lung disease
NSIP: Nonspecific interstitial pneumonia
TBLC: Transbronchial lung cryobiopsies
